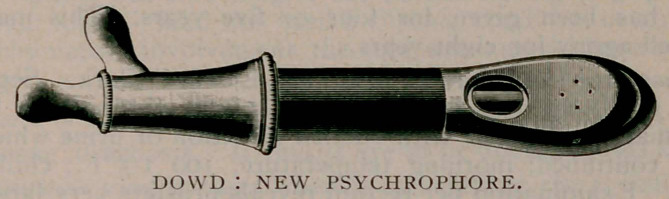# Treatment of Prostatic Disease by Hot Solution

**Published:** 1902-07

**Authors:** J. Henry Dowd

**Affiliations:** Buffalo, N.Y.


### Treatment of Prostatic Disease by Hot Solution.

BY J. HENRY DOWD, M.D., Buffalo, N.Y.

THE beneficial results of heat and cold in the treatment of inflammations need scarcely any mention; yet a word or so may be of value as to the application of these agents, especially the former in musculo-glandular tissue, such as the prostate, where the greatest benefit is to be derived. Until recently when Dr. Guitéras, almost simultaneously with the writer, introduced an instrument which brought the solution in direct contact with the part, all similar applications of heat were made with a closed tube, producing what is known as dry heat. It may be granted that heat was produced, but not that variety that invites or facilitates absorption of inflammatory exudates. Furthermore, where the solution is brought in direct contact with the part we have not only the moist heat, but osmosis takes place, an indispensable requirement in the treatment of these conditions.

On the other hand, and possibly the most important improvement as far as the patient's comfort is concerned, is the application to the tube of hard rubber covering, where it is grasped by the sphincter muscle. This being practically a nonconductor of heat, renders this mode of treatment almost devoid of physical pain to the patient, the one great objection to the psychrophore of Winternitz. Although water alone is very beneficial, much better results are produced and osmotic action favored if some substance is added, preferably salt, such as normal salt solution (a dram to a quart of water) and used at a temperature of 100° to

110°F. Mechanically speaking, heat dilates and a congestion takes place, more blood being brought to the part.

It must now be our aim while the part is returning to its former condition to promote, as far as possible, absorption of the inflammatory exudate; or, plainly speaking, make the

DOWD: NEW PSYCHROPHORE.

returning blood pick up and carry away the intravascular and intracellular exudate. For this reason it is advisable as soon as the tube is withdrawn to insert a suppository, the following being the writer's favorite:

| | | |
|---|---|---|
| ℞ | Ichthyol . . . . . . . . . . . . . . . . . . . . . . . | gtt. ij. |
| | Ext. bellad . . . . . . . . . . . . . . . . . . . . . . | gr. ¼. |
| | Ext. hyoscy . . . . . . . . . . . . . . . . . . . . . . | ~~ʒj~~ gr. i. |
| | Potassi iodide . . . . . . . . . . . . . . . . . . . . | gr. i. |

M. One suppository for the rectum.

Briefly speaking, the following diseased conditions respond most rapidly to moist heat and the use of suppositories: chronic parenchymatous prostatitis; chronic follicular prostatitis; exacerbations of inflammation in hypertrophied conditions; sexual neurasthenia, accompanied by hysterical prostate; chronic inflammation of ejaculatory ducts and vesicles.

The following illustrative cases are presented:

Case I.—Consultation with Dr. J. F. Meyer. July, 1896, A. J., aged 36; married; gives following history: gonorrhea first time in 1885; second, three years latter. Stricture cut by a Buffalo surgeon in 1887. Before gonorrhea seminal emissions in excess; masturbated when young. Now complains of pain above the pubes, fulness and pain in perineum, pain on defecation, shooting pain in testicles, erections cause agony, pain at coitus and ejaculation makes him sick for a week. Urinates normally during the day, once at night and following the stream is a glary fluid. Some pus in urine. Endoscope shows two or three congested spots in the anterior urethra. Examination per rectum shows: prostate enlarged, especially right lobe, vesicles normal. Patient in a marked state of neurasthenia; claims cannot attend to business as he cannot sit or stand for more than half an hour at a time.

The hot solution was ordered used nightly for half an hour at each sitting and suppositories of ichthyol, etc., to be inserted thereafter. Patient put upon arsenauro for nervous system and advised to give up city life and go into the country. Steady and uninterrupted improvement followed, and he reported six months afterward, no pain of any account, erections now cause but very slight pain and coitus pleasurable, without after-effects. The prostate has been normal in size and practically no treatment has been given for four or five years. This man had suffered agony for eight years.

Case II.—Consultation with Dr. Edward Clark. Sept. 28, 1899, J. W., aged 52 years; married; about three weeks before this man was seized with sudden retention of urine which has since continued; morning temperature, 100 1-5°F.; chills last night. Examination per rectum reveals prostate very large and tender, but no perceptible spot of fluctuation. Advised catheterisation every six hours with hot solutions by rectal instrument morning and evening, a suppository of ichthyol, according to formula, to be used after each treatment.

Urine commenced to drip after using the instrument five times, and in twelve hours a good stream could be passed. Previous history showed that he had been obliged to urinate from three to five times nightly for the past four years, but never had an attack of retention before. Urinary examination was as follows: specific gravity, 1015, acid, very opaque, indican increased, albumin, pelvic, bladder and posterior urethral epithelial cells, with plenty of pus cells, which are seen in clumps. A year previous to this the urine had been examined by Dr. Carpenter who found the patient had pyelitis.

Case III.—Consultation with Dr. C. J. Reynolds. February, 1896, B. E., aged 35 years; married. Gonorrhea once in 1881 or 1882, lasting five weeks. In 1895 cut for strictures, being told there were three. Pains over pubes, very nervous; masturbated when young to excess and until a year ago frequent emissions; no trouble with erections and found no obstruction in urethra. Examination per rectum reveals prostate normal, not tender, but right vesicle as large as his thumb. Stripping the vesicle brings away pus in large quantities. Patient was put on tonic treatment with sounds and stripping of the vesicle every five days. Excepting the general improvement no change was noted, the vesicle remaining about the same in size. After this treatment for some time the hot solution was used and almost immediate results were noted, *i.e.*, the vesicle after a couple of weeks became normal in size and in two or three months the patient was discharged well.

This case is reported simply to show the great value of heat or cold when applied to a condition calling for either of these agents:

Case IV.—Consultation with Dr. E. M. Dooley. October, 1896, S. J., aged 43; married. About twelve days after the onset of an acute gonorrhea he noticed that urination was increased to every hour and a half by day and about twenty times at night. Sedatives were used with no result, when the patient was sent to me. The case was diagnosticated as follicular prostatitis and the psychrophore used with ice water, a 1-15,000 solution of silver being thrown into the bladder. Improvement was like magic, the patient having to get up but once that night. Had this been a chronic condition or an acute parenchymatous involvement, the cold water would have been of no avail.

There is but one contraindication to the use of the hot spray—a wrong diagnosis. If results are to be obtained, this instrument must be used in cases where it and nothing else is indicated.

378 FRANKLIN STREET.

## Figures and Tables

**Figure f1:**